# Hybrid Dispersion Model Characterization of PAZO Azopolymer Thin Films over the Entire Transmittance Spectrum Measured in the UV/VIS/NIR Spectral Region

**DOI:** 10.3390/ma15238617

**Published:** 2022-12-02

**Authors:** Dorian Minkov, Lian Nedelchev, George Angelov, Emilio Marquez, Blaga Blagoeva, Georgi Mateev, Dimana Nazarova

**Affiliations:** 1 College of Energy and Electronics, Technical University of Sofia, 2140 Botevgrad, Bulgaria; 2 Institute of Optical Materials and Technologies, Bulgarian Academy of Sciences, 1113 Sofia, Bulgaria; 3 Department of Microelectronics, Technical University of Sofia, 1000 Sofia, Bulgaria; 4 Faculty of Science, Department of Condensed-Matter Physics, University of Cadiz, 11510 Puerto Real, Cadiz, Spain

**Keywords:** innovative dispersion model, PAZO polymer, thin film, optical characterizatics, UV/VIS/NIR transmittance spectra

## Abstract

Notwithstanding the significant optical applicability of PAZO polymer films, there are no accurate data about their optical characteristics. To remedy this shortcoming, in this study three PAZO polymer thin films are characterized, with dissimilar thicknesses, on glass substrates using only one UV/VIS/NIR transmittance spectrum *T*(*λ*) per sample and an original hybrid dispersion model (HDM). HDM is based on the Tauc–Lorentz model, the new amorphous dispersion formula, the Tauc–Lorentz–Urbach model of Foldyna and the Tauc–Lorentz–Urbach model of Rodriguez. HDM with two oscillators is employed in characterizations of the PAZO polymer films in the range [300, 2500] nm, whereby the root-mean-square deviation (*RMSD*) of the fitted transmittance spectrum with respect to *T*(*λ*) does not exceed 1.6 × 10^−3^. Decreasing *RMSD* by 2.3% to 94.4% is demonstrated by employing HDM compared with the above mentioned four popular dispersion models, for each one of the studied films. HDM is applicable to amorphous films independent of their thickness as well as to cases of non-transparent substrate.

## 1. Introduction

Azopolymers are materials used in polarization holography, for light controlled surface nanopatterning, inscription of polarization-selective holographic optical elements and other photonics applications [1,2,3,4]. One widely investigated azopolymer is poly [1-[4-(3-carboxy-4-hydroxyphenylazo)benzenesulfonamido]-1,2-ethanediyl, sodium salt], [-CH_2_CH[NHSO_2_C_6_H_4_N=NC_6_H_3_(OH)CO_2_Na]-]_n_, commonly denoted as PAZO. It is commercially available from Sigma-Aldrich, which allows reproduction and verification of reported data and results.

Over the past two decades, the PAZO polymer has found numerous applications: layer-by-layer (LbL) films containing PAZO prepared and used for second harmonic generation [5,6], studies of photoisomerization [7] and photoinduced birefringence [8,9,10,11]. Recently, an optimal recording wavelength for thin films of PAZO was determined to be *λ* ~ 442 nm, whereby the highest birefringence Δ*n*_max_ = 0.081 was obtained for this wavelength [12]. In addition, the photoinduced birefringence in PAZO has exceptionally high thermal stability to almost 300 °C [13].

Another important advantage of the azopolymer PAZO is its water solubility. Thus, toxic organic solvents, which are usually required for film deposition of other azopolymers, can be avoided. Furthermore, the water solubility of PAZO allows for easy preparation of photoanisotropic nanocomposite layers containing PAZO and nanoparticles such as silver and gold nanoparticles [14,15], goethite nanorods [16], biologically active metal complexes [17], etc. These nanocomposite materials show enhanced photo response, higher birefringence, increased diffraction efficiency and higher surface relief modulation compared with non-doped polymer. For most of its optical applications, the azopolymer PAZO is deposited as thin films with a thickness ranging from several nanometers to several microns [7,12,13,14,15,16,17,18,19].

Designing efficient photonics devices including PAZO polymer thin films requires accurate knowledge of their main spectral characteristics in the UV/VIS/NIR region, such as the refractive index *n*(*λ*) and the extinction coefficient *k*(*λ*). Data about *n*(*λ*) and *k*(*λ*) of doped PAZO polymer thin films have been derived by solving a system of two equations, for the transmittance spectrum *T*(*λ*) and the reflectance spectrum *R*(*λ*) of the film on a glass substrate, with respect to the unknowns *n*(*λ*) and *k*(*λ*) [20,21]. However, the somewhat wavy or spiky appearance of *n*(*λ*) and/or *k*(*λ*) from [20,21] exemplifies a general problem of insufficiently accurate simultaneous computation of *n*(*λ*) and *k*(*λ*) from *T*(*λ*) and *R*(*λ*) due to difference in the light spots on the film surface in these two measurements.

Furthermore, it is known that relatively accurate optical characterization of a thin film can be achieved via analysis only of the normal incidence UV/VIS/NIR transmittance spectrum *T*(*λ*) of the film on a light transmitting substrate such as glass or quartz [22,23]. Notably, the appearance of *T*(*λ*) depends strongly on the main film thickness characteristics: the average thickness $\bar{d}$ over the light spot on the film surface and the thickness non-uniformity ∆d over this light spot [24].

The transmittance of light through a thin film on a thick substrate was formulated in [25]:

(1) $$T{\left(\lambda \right)}_{}{=}_{}\frac{{\left(\tau_{a,f}\tau_{f,s}\tau_{s,a}\right)}^{2}x_{s}}{\varphi_{2}-\varphi_{1}}\int_{\varphi_{1}}^{\varphi_{2}}{\frac{x_{}d\varphi }{a_{1}-b_{1}cos\left(\varphi \right)+c_{1}sin\left(\varphi \right)}}_{},$$

where

$$\varphi =4\pi nd/\lambda {,}_{}\varphi_{1}=4\pi n(\bar{d}-\Delta d)/\lambda {,}_{}\varphi_{2}=4\pi n(\bar{d}+\Delta d)/\lambda {,}_{}x=exp(-4\pi kd/\lambda ){,}_{}$$

$$x_{s}{=}_{}exp(-4\pi k_{s}d_{s}/\lambda ){,}_{}a_{1}{=}_{}1-{\left(\rho_{a,f}\rho_{s,a}xx_{s}\right)}^{2}+\rho_{f,s}{}^{2}(\rho_{a,f}{}^{2}x^{2}-\rho_{s,a}{}^{2}x_{s}{}^{2}),$$

$$b_{1}=2\rho_{a,f}\rho_{f,s}x_{}\left[cos\Delta_{1}-{\left(\rho_{s,a}x_{s}\right)}^{2}cos\Delta_{2}\right]{,}_{}c_{1}=2\rho_{a,f}\rho_{f,s}x_{}\left[sin\Delta_{1}-{\left(\rho_{s,a}x_{s}\right)}^{2}sin\Delta_{2}\right],$$

$$\tau_{a,f}\tau_{f,s}\tau_{s,a}=\frac{8}{\sqrt{{\left(n+1\right)}^{2}+k^{2}}}\sqrt{\frac{n^{2}+k^{2}}{{\left(n+n_{s}\right)}^{2}+{\left(k+k_{s}\right)}^{2}}}\sqrt{\frac{n_{s}{}^{2}+k_{s}{}^{2}}{{\left(n_{s}+1\right)}^{2}+k_{s}{}^{2}},}$$

$$\rho_{a,f}=\sqrt{\frac{{\left(n-1\right)}^{2}+k^{2}}{{\left(n+1\right)}^{2}+k^{2}}},\rho_{f,s}=\sqrt{\frac{{\left(n-n_{s}\right)}^{2}+{\left(k-k_{s}\right)}^{2}}{{\left(n+n_{s}\right)}^{2}+{\left(k+k_{s}\right)}^{2}}},\rho_{s,a}=\sqrt{\frac{{\left(n_{s}-1\right)}^{2}+k_{s}{}^{2}}{{\left(n_{s}+1\right)}^{2}+k_{s}{}^{2}}},$$

$$\begin{array}{l} \Delta_{1}=tan^{-1}\left(\frac{2k}{n^{2}+k^{2}-1}\right)+tan^{-1}\left[\frac{2(kn_{s}-k_{s}n)}{n^{2}-n_{s}{}^{2}+k^{2}-k_{s}{}^{2}}\right],\\ \Delta_{2}=tan^{-1}\left(\frac{2k}{n^{2}+k^{2}-1}\right)-tan^{-1}\left[\frac{2(kn_{s}-k_{s}n)}{n^{2}-n_{s}{}^{2}+k^{2}-k_{s}{}^{2}}\right], \end{array}$$

*x*(*λ*) is the absorbance of the film, as the subscripts “f”, “s” and “a” refer to the film, the substrate and the air, respectively. It was assumed in the derivation of Equation (1) that the film thickness *d* has a continuous uniform distribution in the interval [$\bar{d}$ − ∆d, $\bar{d}$ + ∆d] over the light spot, whereby the light passing through the film is considered to be coherent. The light passing through the substrate is regarded as non-coherent, due to the significant thickness of the substrates used in practice, thus quenching light interference there [24,25].

The main goal of this paper is to characterize accurately amorphous PAZO polymer thin films, with dissimilar thicknesses, over the entire *T*(*λ*) measured in the UV/VIS/NIR spectral region. To enhance the characterization accuracy, an innovative hybrid dispersion model, abbreviated as HDM, is proposed for amorphous materials. Increasing accuracy of characterization of PAZO films is demonstrated using HDM in comparison with arguably the most popular dispersion models for amorphous materials. Utilizing HDM, some PAZO polymer film characteristics are established, for the first time, such as absorption edge *E*_c_, whereas for *E* > *E*_c_ prevails absorption due to interband electronic transitions over absorption due to localized electronic states, regions of photon energies *E* > *E*_c_ associated with contributions from indirect allowed and direct forbidden interband transitions, validity of the Urbach approximation and Urbach energy determining the absorption for *E* < *E*_c_, etc.

## 2. Materials and Methods

### 2.1. Preparation of the Samples and Measurement of Their Transmittance Spectra T(λ)

The polymer films are spin-coated from solution of the azopolymer PAZO on 1.65 mm thick BK7 glass substrates. Three such samples, denoted as D1, T2 and M1, are studied. The thicker films D1 and T2 are spin-coated at 1500 rpm from water solutions with PAZO concentrations 150 mg/mL and 135 mg/mL, respectively. The significantly thinner film M1, designed not to be extremely thin, is spin-coated using methanol with 25 mg/mL PAZO concentration as solvent, since it has lower viscosity than water and a lower spin rate of 1000 rpm. Transmittance spectra *T*(*λ*) of the samples are measured in the spectral range *λ* = [300, 2500] nm using a Cary 05E UV/VIS/NIR spectrophotometer of Varian with 2 nm spectral bandwidth, 1 nm data collection step and 7 mm × 4 mm light spot on the illuminated surface.

### 2.2. Common Techniques for Characterization of Thin Films Only from T(λ)

Undoubtedly, highest accuracy of computation of $\bar{d}$ can be achieved by employing the optimizing envelope method for *T*(*λ*) (OEMT), which is a model free method applicable only to relatively thick films with *T*(*λ*) exhibiting at least five apparent extrema [26]. OEMT is based on external smoothing of *T*(*λ*) to account for the partial coherence of light in the film, double transformation of *T*(*λ*) to exclude the substrate absorption and utilization of a part of *T*(*λ*) providing the lowest fluctuation of estimates of $\bar{d}$ for the extrema of *T*(*λ*). Our studies of a-Si and As_x_Te_1−x_ films showed that the error in computation of their $\bar{d}$ derived from OEMT does not exceed 0.1% [27,28].

Optical characterization of a thin film with arbitrary thickness, over the entire range of its UV/VIS/NIR spectrum *T*(*λ*), requires using optical dispersion model (DM) formulating both *n*(*λ*) and *k*(*λ*) (explicitly or by means of the complex dielectric function $\dot{\varepsilon }$). However, DMs are based on assumptions which can be inaccurate for some thin films. Arguably, the most popular DMs of amorphous materials in the UV/VIS/NIR range are: the Tauc–Lorentz model (TL) [29,30], the new amorphous dispersion formula (NADF) [31,32], the Tauc–Lorentz–Urbach model of Foldyna (TLUF) [33,34], and the Taucv–Lorentz–Urbach model of Rodriguez (TLUR) [35,36]. Importantly, TL and NADF assume *k*(*λ*) = 0 for *E* < *E*_g_, where *E*(eV) = 1239.8/λ(nm) is the photon energy and *E*_g_ is the optical bandgap, which can lead to inaccurate characterization of amorphous films in the range *E* < *E*_g_. On the other hand, TLUF and TLUR assume existence of an Urbach tail (i.e., *α* =$\alpha_{0}$ exp(*E*/*E*_U_) where *E*_U_ is Urbach energy) in the range *E* < *E*_c_ > *E*_g_. However, TLUF and TLUR are one oscillator models, which can result in incorrect characterization of films, with different kinds of electron interband transitions, in the range *E* > *E*_c_. For all four DMs discussed in this paragraph, the film characteristics *n*(*λ*), *k*(*λ*), $\bar{d}$ and ∆d can be determined by fitting the transmittance spectrum calculated from Equation (1) to the measured spectrum *T*(*λ*).

In addition to DMs including both *n*(*λ*) and *k*(*λ*), there are DMs formulating only one of them (explicitly or implicitly). Such DMs can provide asymptotic representation of *n*(*λ*) or *k*(*λ*). For instance, for photon energies *E* → 0, the refractive index of amorphous materials can be approximated using the Wemple–DiDomenico relationship representing an undamped single oscillator [37,38]:

(2) $${\frac{1}{n^{2}-1}}_{}{\overset{E\to 0}{\to }}_{}\frac{E_{0}}{E_{d}}-\frac{E^{2}}{E_{0}E_{d}},$$

where *E*_0_ and *E*_d_ are the energy and the strength of the oscillator. Accordingly, the Wemple–DiDomenico plot depicts the dependence of (*n*^2^ − 1)^−1^ as a function of *E*^2^ [38].

In addition, for *E* < *E*c > *E*g, the absorption coefficient α = 4π*k*/λ of amorphous materials can be described using the Urbach rule corresponding to structural disorder generating localized electronic states and the Urbach tail [39,40]:

(3) $$\log_{10}(\alpha )\overset{E<E_{c}>E_{g}}{\to }\log_{10}(\alpha_{0})+\frac{1}{\ln(10)E_{U}}E_{}.$$

Therefore, the Urbach plot usually represents log_10_(α) versus *E* [40].

Furthermore, the absorption of photons not too far above the absorption edge of amorphous materials is associated with electron transitions from the valence band to the conduction band, as α can be approximated via the Tauc rule:

(4) $${\left(\alpha E\right)}^{1/p}\overset{E>E_{c}}{\to }A_{1}\left(E-E_{g}\right),$$

where *p* = 2 for indirect allowed transitions, *p* = 3/2 for direct forbidden transitions, *p* = 1/2 for direct allowed transitions and *p* = 3 for indirect forbidden transitions [41,42]. Correspondingly, the Tauc plots show (α*E*)^1/*p*^ as a function of *E* [42]. Notably, Equations (2)–(4) can be depicted as straight lines in their Wemple–DiDomenico plot, Urbach plot and Tauc plot, respectively [43,44].

Moreover, Equation (4) has been reformulated as follows:

(5) $$\log_{10}\left(\alpha E\right)\overset{E_{g}+2(eV)>E>E_{c}}{\to }A_{2}+pE$$

using Taylor series expansion [45]. According to Equation (4) and (5), the predominant type of interband transitions is determined using the value of *p*, which represents the slope of a straight line depicting the dependence of log_10_(α*E*) versus *E* within the interval [*E*_c_, *E*_g_ + 2 (eV)].

On the other hand, the following approximation of *n*(*E*) was developed recently based on Equation (2) [46]:

(6) $$n^{2}(E)\overset{E>E_{g}}{\to }1+\left[n^{2}(0)-1\right]\times \left\{1+\frac{E}{4\left(E_{M}-E_{g}\right)}\ln\frac{\left[2E_{M}-\left(E_{g}+E\right)\right]\left(E_{g}+E\right)}{\left[2E_{M}-\left(E_{g}-E\right)\right]\left(E_{g}-E\right)}\right\},$$

as *n*(0) = *n*(*E* = 0) is the static refractive index and *E*_M_ is the energy difference between the energy centers of the conduction band and the valence band, whereby these bands are assumed to have the same widths. Provided that *n*(*λ*) and *E*_g_ are known, Equation (6) can be used to compute an approximated value of *E*_M_.

The accuracy of every characterization of a thin film in the spectral range *λ* ⊂ [*λ*_1_, *λ*_2_], only from *T*(*λ*), is related to the figure of merit *F* defined as:

(7) $$F=1000\times RMSD\left(T_{c},T\right){=}_{}1000\times {\sqrt{\frac{\sum_{\lambda_{1}}^{\lambda_{2}}{}_{}{\left[T\left(\lambda \right)-T_{c}\left(\lambda \right)\right]}^{2}}{\left(\lambda_{2}-\lambda_{1}+1\right)}}}_{}\geq 0,$$

where the computed transmittance spectrum *T*_c_(*λ*) is derived by replacing the computed values of *n*(*λ*), *k*(*λ*), $\bar{d}$ and ∆d in Equation (1). Notably, smaller value of *F* corresponds to more accurate thin film characterization since *F* is proportional to the root mean square deviation *RMSD*(*T*_c_,*T*) of *T*_c_(*λ*) from the measured spectrum *T*(*λ*).

### 2.3. Hybrid Dispersion Model for Characterization of Amorphous Thin Films Only from T(λ)

As already mentioned, each one of the dispersion models TL, NADF, TLUF and TLUR is based on assumptions which can be inaccurate in the UV/VIS/NIR region for some amorphous films. More specifically, TL and NADF can be inaccurate in the range *E* < *E*_c_, while TLUF and TLUR can be inaccurate in the range *E* > *E*_c.,_ as described in Section 2.2. Aiming at resolution of this drawback, in this paper is compiled and employed a hybrid dispersion model (named here HDM) using only the above four DMs.

At stage 1 of HDM are computed approximations of *n*(*λ*), *k*(*λ*), $\bar{d}$ and ∆d via execution of fits using TL, NADF, TLUF and TLUR over the entire measured spectrum *T*(*λ*). DM providing the lowest *F* over the entire spectrum, amongst the last four DMs, is selected, after which $\bar{d}$ and ∆d computed from the fit using this DM are regarded as $\bar{d}$ and ∆d of the film. At stage 2 of HDM, *F* is calculated in the energy intervals [min(*E*), *E*c] and [*E*_c_, max(*E*)], for the above four DMs, whereby *E*c for TL and NADF is taken from TLUF or TLUR with a smaller *F* at stage 1. Then, wavelength range (with one boundary corresponding to *E*c and the other boundary being either min(λ) or max(λ)) where the DM selected in stage 1 provides the lowest *F* is established and the DM providing lowest *F* in the rest of the measured spectrum is determined. At stage 3 of HDM, fit using the DM determined at the end of stage 2 is performed, however, only over the above mentioned rest of the measured spectrum, by employing the already fixed $\bar{d}$ and ∆d. *n*(*λ*) and *k*(*λ*), computed by HDM, consist of their values obtained by the DM selected at stage 1 over the wavelength range where it provides the lowest *F* (determined at stage 2) and their values over the rest of the measured spectrum (obtained at stage 3).

## 3. Results

**Figure 1:**
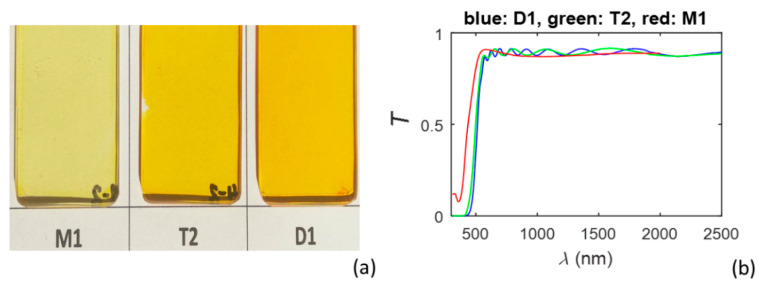
(**a**) Photos of the PAZO polymer samples; (**b**) Measured transmittance spectra of these samples.

Photos of the studied three PAZO polymer film on substrate samples are shown in Figure 1a, and their measured transmittance spectra *T*(*λ*) are presented in Figure 1b. The refractive index *n*_s_(*λ*) and the extinction coefficient *k*_s_(*λ*) of the BK7 glass substrates are taken from [47].

### 3.1. Results from Characterizations of the PAZO Polymer Films Using HDM and T(λ)

The PAZO polymer films are characterized, using HDM, only from *T*(*λ*) of the samples. With respect to this, it is seen from Figure 1b that *T*(*λ*) of the sample M1 includes absorption around *λ* ~ 350 nm, which is below *λ* for the absorption edge. Accordingly, the HDM characterizations employ two oscillators. Values of the DM parameters regarding *n*(*λ*) and *k*(*λ*), obtained via the HDM characterizations of these films, are shown in Table 1. In Table 1, *f*_i_ is the strength of the “i”-th oscillator, *E*_i_ is its central energy and *B*_i_ is its energy broadening, as the subscript “1” refers to the lower energy oscillator and the subscript “2” to the higher energy oscillator. Moreover, *E*_g0_ is the fitted bandgap, which can differ from the optical bandgap *E*_g_, as discussed for rf-magnetron-sputtered a-Si in [34], and *ε*_r_(∞) = real[$\dot{\varepsilon }$(*E* → ∞)] = *n^2^*(*E* → ∞).

**Figure 2:**
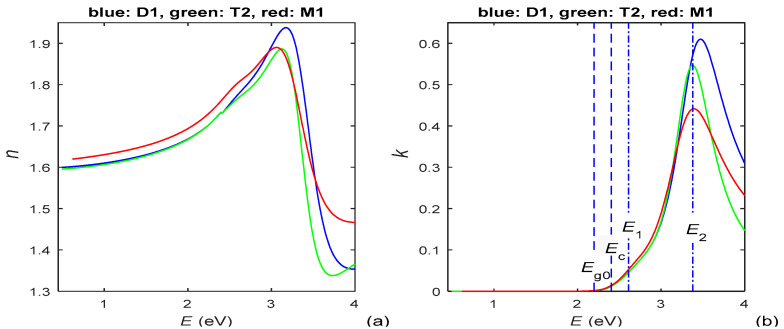
(**a**) Refractive indices of the PAZO polymer films; (**b**) extinction coefficients of the same films. Some important energies for the film D1, among the data in Table 1, are illustrated by vertical blue lines.

Spectral dependencies *n*(*E*) and *k*(*E*) of the studied PAZO polymer films, computed using HDM and *T*(*λ*) from Figure 1b, are presented in Figure 2.

**Figure 3:**
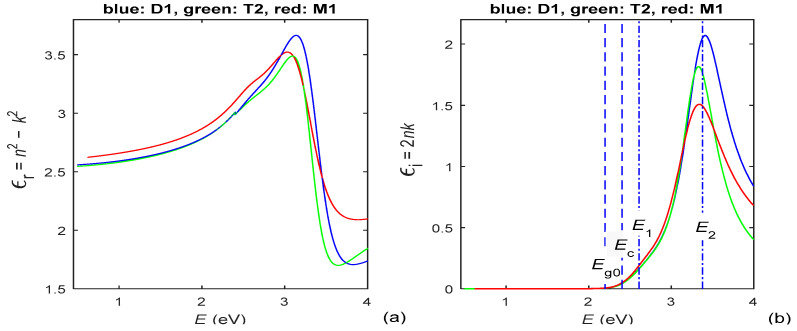
(**a**) The real part of the complex dielectric function of the PAZO polymer films; (**b**) the imaginary part of the complex dielectric function of the same films.

The real part *ε*_r_(*E*) and the imaginary part *ε*_i_(*E*) of the complex dielectric function $\dot{\varepsilon }$ of the same films are derived from the data depicted in Figure 2; hence, *ε*_r_(*E*) and *ε*_i_(*E*) are shown in Figure 3.

### 3.2. Characteristics of the PAZO Polymer Films Derived from HDM Results and DMs for n(λ) or k(λ)

As discussed in Section 2.2, Equations (2)–(6) represent DMs of amorphous materials including, explicitly or implicitly, either *n*(*λ*) or *k*(*λ*). Correspondingly, it can be established, from the Wemple–DiDomenico plot, Urbach plot and Tauc plot, whether *n*(*λ*) and *k*(*λ*) computed using HDM and *T*(*λ*) satisfy Equations (2)–(4). Furthermore, the film parameters included in these DMs can be determined by means of replacing these *n*(*λ*) or *k*(*λ*) in Equations (2)–(6).

**Figure 4:**
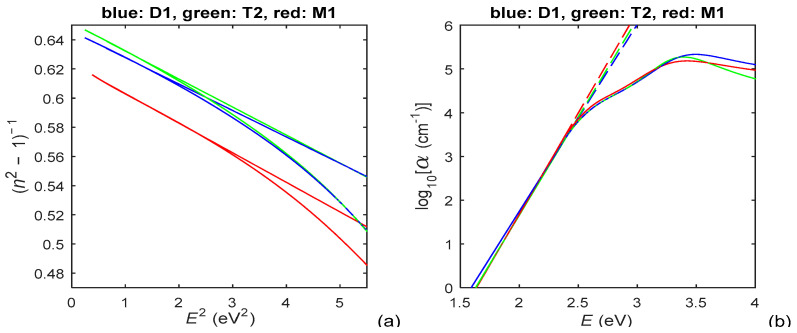
(**a**) Wemple–DiDomenico plot, utilizing *n*(*E*) from Figure 2a, for the PAZO polymer films; (**b**) Urbach plot, utilizing *k*(*E*) from Figure 2b, for the same films.

With respect to the above, the Wemple–DiDomenico plot and Urbach plot, prepared by utilizing *n*(*E*) and *k*(*E*) from Figure 2, are shown in Figure 4.

**Figure 5:**
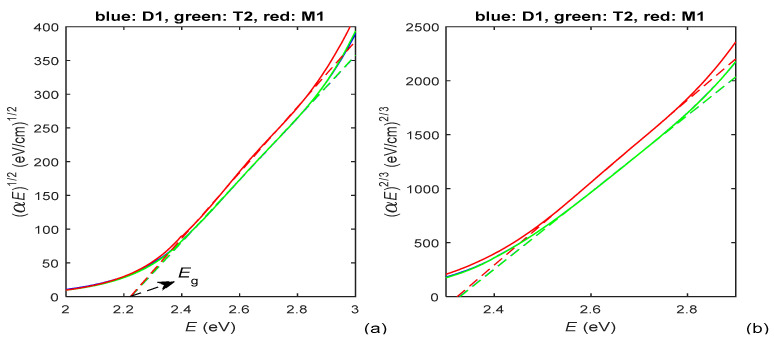
(**a**) Tauc plot for *p* = 2, utilizing *k*(*E*) from Figure 2b, for the PAZO polymer films; (**b**) Tauc plot for *p* = 3/2, utilizing *k*(*E*) from Figure 2b, for the same films. The straight line approximations are depicted by dashed lines.

According to the data about *E*_c_ from Table 1, the absorption edge is at *E*_c_ ≈ [2.23, 2.4] eV for the PAZO polymer films. As seen from Equation (5), *p* represents the slope of the linear part of the dependence log_10_(α*E*) versus *E* above the absorption edge, whereby this dependence looks similar to the one from Figure 4b. Based on the above, it is determined, from the chart of log_10_(α*E*) versus *E* and Equation (5), that *p* ⊂ [1.76, 1.84] for photon energies *E* ⊂ [2.7, 3.2] eV. Since these values of *p* satisfy the inequalities 2 > *p* > (2 + 3/2)/2, it is concluded from Equation (4) that the prevailing interband transitions are indirect allowed with *p* = 2 (which is typical for amorphous materials [42,48,49]); however, there is significant contribution from direct forbidden transitions with *p* = 3/2. Therefore, Tauc plots for *p* = 2 and *p* = 3/2, prepared by utilizing *k*(*E*) from Figure 2b, are presented in Figure 5. As per Equation (4), the optical bandgap *E*g equals the photon energy *E* corresponding to the interception point of the straight line approximation of (α*E*)^1/2^, from the Tauc plot in Figure 5a, and the *E*-axis [34,42].

The energy difference *E*_M_ between the centers of the conduction band and the valence band is computed from Equation (6) by utilizing *n*(*E*) from Figure 2a and *E*_g_ derived from the Tauc plot in Figure 5a. The values of film parameters included in the DMs only for *n*(*λ*) or *k*(*λ*), calculated from Equation (2)–(6) as described in this Section, are presented in Table 2.

### 3.3. Results about the Thickness Characteristics and the Accuracy of the Film Characterizations

As mentioned in Section 2.2, our group has accomplished computation of $\bar{d}$ with errors ≤ 0.1% by means of OEMT, which takes into account the substrate absorption. This was achieved for a-Si films with significant localized states absorption revealed by large Urbach energy *E*_U_ ≈ 250 meV and As_x_Te_1−x_ films with much smaller localized states absorption and *E*_U_ ≈ 190 meV, whereby the error in $\bar{d}$ decreases with decreasing *E*_U_ [28]. Since *E*_U_ = [93, 102] meV for the PAZO polymer films studied here, as seen from Table 1, $\bar{d}$ derived from OEMT can be used as a reference value for estimating the accuracy of $\bar{d}$ computed using a particular DM.

Based on the above, $\bar{d}$ of the thicker PAZO polymer films D1 and T2 (only their *T*(*λ*) have at least five apparent extrema) is also derived from OEMT. The computed thickness characteristics $\bar{d}$ and ∆d of the PAZO polymer films and the figure of merit *F*, determining the accuracy of a particular film characterization, are presented in Table 3 for all film characterizations.

Taking into account the comments from the first two paragraphs of Section 3.3, the two pieces of data in red from the fifth column of Table 3 point out that the relative error in computation of $\bar{d}$ using HDM does not exceed 0.6% for the films D1 and T2. In addition, the data in blue from the last column indicate that the figure of merit *F* reduces by [2.3, 17.5]% at the end of HDM characterization, for the three PAZO polymer films, compared to the end of stage 1 of HDM.

## 4. Discussion

As seen from column 2 of Table 1, HDM, used in PAZO polymer films characterization, is based on TLUF for *E* < *E***_c_** and either NADF with two oscillators or TL with two oscillators for *E* ≥ *E***_c_**. This resolves the principal problem of TLUF being one oscillator DM (revealed for *E* ≥ *E***_c_** in cases of multiple oscillators) as well as the principal problem of TL and NADF ignoring absorption by localized electronic states (revealed for *E* < *E***_c_**). In addition, the data from the last two columns of Table 3 show that the smallest value of *F* is achieved for the characterizations using HDM, compared to those using TLUF, TLUR, TL and NADF, as *RMSD*(*T*_c_,*T*) < 1.6 × 10^−3^ for the HDM characterizations. On the other hand, in our recent study of a-Si films, *RMSD*(*T*_c_,*T*) = [4.8, 7.6] × 10^−3^ for TLUR characterizations of a-Si films and [5.5, 12.7] × 10^−3^ for TLUF characterizations of the same a-Si films was accomplished [36]. Importantly, although HDM employs two different DMs, above and below *E***_c_**, no significant discontinuity is observed in the spectral dependencies *n*(λ), *k*(λ), *ε*_r_(*λ*) and *ε*_i_(*λ*), for all studied PAZO polymer films, as seen from Figure 2 and Figure 3. All data discussed in this paragraph indicate high accuracy of the HDM characterizations of PAZO polymer films reported here.

The larger *n*(*E* > *E***_c_**) of the film M1 is attributed to the faster evaporation of its methanol solvent, compared to the water solvent for the films D1 and T2, which leads to a negligible amount of solvent in the film M1 and its larger *n*. In addition, the existence of quasi-linear parts of the solid curves from Figure 4 and Figure 5, depicted by dashed lines, indicate that the PAZO polymer films obey the Wemple–DiDomenico relationship, the Urbach rule and the Tauc rule, which is typical for amorphous materials. Furthermore, it is concluded, based on the data from Figure 5, Equation (4) and comments from [45], that the light absorption is mainly due to indirect allowed transitions for *E* = [2.4, 2.8] eV, whereby there are also significant direct forbidden transitions for *E* = [2.55, 2.75] eV.

In order to compare optical parameters of different PAZO polymer-based films, data from [20,21] and this study are shown in Table 4.

It is seen from Table 4 that the main results from this paper are commensurate with those for doped and spin-coated films from [20]; however, the doped and electrospray deposited films from [21] have a significantly smaller refractive index *n*(*λ*) and optical bandgap *E*_g_.

To the best of our knowledge, the results reported here about PAZO polymer films represent the first characterization of such films only from *T*(*λ*) and their first study using DMs with 2 oscillators. In general, using both HDM and Equation (1) should provide accurate characterization of different kinds of amorphous thin films independent of their thicknesses, including cases of non-transparent substrate.

## 5. Conclusions

The smallest value of *RMSD*(*T*_c_,*T*) = [0.94, 1.59] × 10^−3^ achieved when using HDM compared to TLUF, TLUR, TL and NADF indicates that the characterizations of the studied PAZO polymer films via HDM are more accurate than those using the four arguably most popular DMs for amorphous films. It is established, using HDM, that *n*(*λ*) and *k*(*λ*) of these films in the UV/VIS/NIR spectral region are described correctly by employing two oscillators. As far as we are aware, the absorption edge, the regions of photon energies associated with indirect allowed and direct forbidden interband transitions, the Urbach energy and the energy difference between the centers of the conduction band and the valence band are determined for the first time for PAZO polymer films. In the future, we intend to characterize polymer films prepared using different technology and doped PAZO polymer films, as well as other types of amorphous films, using HDM.

## Figures and Tables

**Table 1:** Values of the parameters, determining *n*(*λ*) and *k*(*λ*) of the PAZO polymer films, obtained using HDM and *T*(*λ*). The dispersion models selected at stages 1 and 2 of HDM are described in the second column, whereby the symbol “.2osc” denotes that the respective DM employs two oscillators.

Film	HDM for min(*E*) ≤ *E* < *E*_c_, HDM for *E*_c_ ≤ *E* ≤ max(*E*)	*E*_g0_ (eV)	*E*_c_ (eV)	*E*_U_ (meV)	*f*_1_ ×0.001	*E*_1_ (eV)	*B*_1_ (eV)	*f*_2_ ×0.001	*E*_2_ (eV)	*B*_2_ (eV)	*ε*_r_ (∞)
D1	TLUF, NADF.2osc	2.20	2.40	101.5	15.2	2.61	0.36	36.6	3.37	0.34	2.27
T2	TLUF, TL.2osc	2.13	2.40	96.8	2154	2.67	0.72	7061	3.31	0.54	2.35
M1	TLUF, NADF.2osc	2.02	2.23	93.8	9.12	2.62	0.30	37.7	3.27	0.39	2.43

**Table 2:** Values of parameters of the PAZO polymer films, included in the DMs only for *n*(*λ*) or *k*(*λ*), calculated from Equation (2)–(6).

Film	*E*_0_ (eV)	*E*_d_ (eV)	*n*(0)	*E*_g_ (eV)	*p*	*E*_M_ (eV)
D1	5.97	9.25	1.60	2.225	1.77	6.30
T2	5.83	8.94	1.59	2.224	1.84	6.09
M1	5.55	8.90	1.61	2.223	1.76	5.78

**Table 3:** Computed values of the thickness characteristics  d ¯ and ∆d of the PAZO polymer films and the figure of merit *F* for all performed characterizations. The average film thickness over the light spot derived from OEMT is denoted by “$\bar{\mathrm{do}}$” and the figure of merit for HDM characterization by “*F*_HDM_”. The quantity in the last column represents the reduction of the figure of merit *F* when using HDM compared to using the other four DMs. The data acquired via DM selected at stage 1 of HDM are typed on a grey background and the data regarding the entire HDM are in red.

Film, $\bar{do}\;(\mathrm{nm})$	Dispersion Model	Δd (nm)	$\bar{d}$ (nm)	$(\bar{d}-\bar{do}$ $)/\bar{do}\;(\%)$	*F*	(*F*−*F*_HDM_)/*F* (%)
D1, 1693	TLUF	32.2	1684	−0.555	1.63	**2.3**
	TLUR	32.3	1684	−0.573	1.78	10.4
	TL.2osc	33.8	1682	−0.673	2.99	46.8
	NADF.2osc	33.5	1682	−0.685	2.34	31.9
	HDM	32.2	1684	−0.555	*F*_HDM_ = 1.59	
T2, 992.4	TLUF	31.9	988.3	−0.413	1.14	**17.5**
	TLUR	31.3	985.8	−0.665	1.52	38.0
	TL.2osc	36.4	986.3	−0.614	2.14	94.4
	NADF.2osc	31.4	986.7	−0.574	1.47	35.6
	HDM	31.9	988.3	−0.413	*F*_HDM_ = 0.944	
M1, -	TLUF	0.0	162.2		4.40	74.8
	TLUR	0.0	163.7		14.9	92.6
	TL.2osc	30.3	157.2		1.49	40.1
	NADF.2osc	25.6	159.6		1.15	**3.3**
	HDM	25.6	159.6		*F*_HDM_ = 1.11	

**Table 4:** Computed optical parameters of PAZO polymer films. The films from [20] have been doped with [1, 5] wt% particles of Cu(II) 3-amino-5,5′-dimethylhydantoin and spin-coated, whereas the films from [21] have been doped with [0, 20] mol% particles of (GeTe_4_)_100−x_Cu_x_ and electrospray deposited.

Parameter	*n*	*k*	*n*	*k*	*n*	*k*	*E*_g_ (eV)
From	*λ* = 440 nm		*λ* = 1000 nm		*λ* = 2500 nm		
[20]	[1.85, 1.9]	[0.07, 0.075]	[1.56, 1.64]	[0.003, 0.014]	[1.45, 1.5]	[0.01, 0.02]	[2.45, 2.6]
[21]	[1.22, 1.42]	[0.024, 0.06]	[1.26, 1.45]	[0.01, 0.06]	-	-	[1.35, 1.75]
this study	[1.80, 1.85]	[0.09, 0.105]	[1.61, 1.64]	[1.1, 1.5]×10^−7^	[1.59, 1.60]	0	[2.22, 2.23]

## Data Availability

Data regarding this article will be shared on request.
